# Study on dust control technology of mobile spray combined with full-section fog curtain in return airway of header working face

**DOI:** 10.1371/journal.pone.0277710

**Published:** 2022-11-17

**Authors:** Deji Jing, Hongwei Liu, Tian Zhang, Shaocheng Ge, Zhuo Jiang, Qiang Zhang

**Affiliations:** 1 College of Safety Science and Engineering, Liaoning Technical University, Fuxin, China; 2 Research Institute of Safety Science and Engineering, Liaoning Technical University, Fuxin, China; 3 Thermodynamic Disasters and Control of Ministry of Education, Liaoning Technical University, Fuxin, China; 4 Safety and Emergency Management Engineering College, Taiyuan University of Technology, Taiyuan, China; 5 Liaoning Tiefa Energy Co., Ltd, Tieling, China; Tongji University, CHINA

## Abstract

For the problem of coal dust pollution in the return air lane of the comprehensive mining working face of soft rock mines.Based on the principle of supersonic siphon pneumatic atomization dust control, mobile vehicle-mounted pneumatic spraying combined with full-section fog curtain dust control technology is proposed to address the coal dust pollution problem in the return air tunnel of the comprehensive mining working face of soft rock mines. This technology has a wider spraying range, stronger wind resistance and lower energy consumption.Using the k-ε turbulence module and the fluid flow particle tracking module of COMSOL simulation software, a three-dimensional numerical model of the return air tunnel was established. The effect of wind flow characteristics on the diffusion range of coal dust and fog droplets was analysed, and the dust transport pattern and dust control effect of the new technology were obtained for different cross-sectional return airways. The results show that the velocity of the wind flow is continuously decayed by the slope, and the dust of different particle sizes is distributed differently by the inertial force. Coal dust with particle sizes larger than 6.5 μm accumulates below the structure at a lower velocity, and coal dust with particle sizes smaller than 4.5 μm is mostly suspended above the structure at a higher velocity. The device effectively stops the transport of dust and covers the whole section of the roadway, and the dust removal efficiency reaches 96.53%~97.93%, which provides relevant theoretical support and treatment means for the control of dust pollution in the return airway of coal mines.

## 1. Introduction

**Fig 1:**
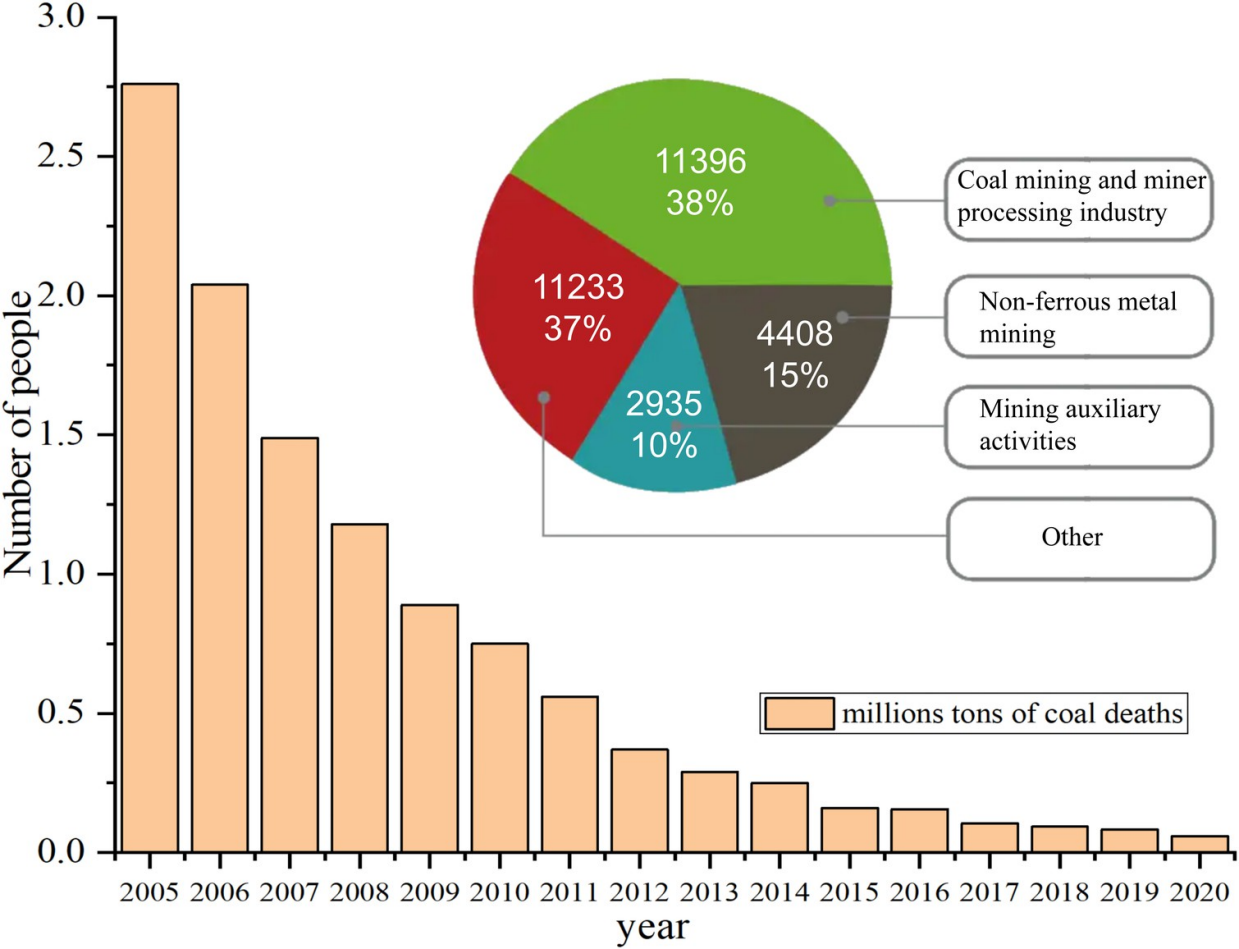
Mining accident fatality rate per million tonnes of coal and pneumoconiosis composition, 2005 to 2020.

In underground coal mines, the amount of dust generated during coal mining operations continues to rise as mechanization increases, posing a significant health risk to workers [1–7]. In addition, there is a risk of combustion when the dust content in the air reaches a certain concentration. Despite the installation of internal and external sprays or other dust reduction measures on coal mining machines, much dust, especially fine dust, is still inhaled into the lungs of workers [8–13]. There are many industries with dust hazards in China, and dust explosion accidents and occupational pneumoconiosis have long been at a high incidence [14–19]. According to the data, the total number of fatalities in coal production accidents, from approximately 120,000 to less than 60,000 between 2005 and 2020, and the number of fatalities per million tonnes of coal from 2.7 to approximately 0.059, the large reduction in the total number of fatalities in coal production accidents indicates that China’s mine safety production has been improving year by year and has developed significantly; however, the number of mine pneumoconiosis cases still accounts for 62.52% of the total number of new pneumoconiosis cases in the industry. The main reason for the high number of pneumoconiosis cases in mines is the lack of attention given to occupational pneumoconiosis in China’s coal and other enterprises and the poor treatment of respiratory dust generated during coal production. The composition of the mortality rate and pneumoconiosis for mining accidents per million tonnes of coal in China from 2005 to 2020 is shown in Fig 1.

At present, many solutions have been proposed and applied to implement the dust pollution problem at the comprehensive mining face, resulting in improvements in the working environment at the comprehensive mining face [20–24]. Cheng et al. studied a multidirectional cyclonic air curtain dust suction control device and optimized the formation mechanism of the wind curtain and the optimal wind curtain dust control parameters [25]. Paine et al. simulated different phases of the cyclonic nozzle spraying process and analysed the effect of different pressures in distinct phases on the nozzle range produced [26]. At present, spray atomisation technology in the world mainly focuses on nozzle atomisation characteristics, and dust pollution control technology in return airways has not yet formed a complete system [27,28]. Most coal mines set up fog curtains in the return airway, spraying water mist to form a water curtain to block the diffusion of dust, however, many mines form soft coal rock structures due to the high water content of the coal seam, resulting in serious deformation of the roof and sidewalls of the roadway and narrowing roadway space as well as large changes in the roadway cross-section; thus, it is impossible to install fixed fog curtain supports, and it is difficult to effectively solve the problem of dust pollution in the return airway with a single fog curtain to reduce dust [29,30] due to the unsatisfactory nozzle atomisation effect and susceptibility to the influence of the tunnel wind flow [31,32]. The wind flow velocity in the return airway is large, and it is difficult to effectively combine ordinary fog screen droplets and dust under strong disturbance wind flow conditions [33,34]. The respiratory dust concentration in the return airway is high, and ordinary mining nozzles have poor atomization effects with large droplet particle sizes and uneven dispersion, resulting in poor dust reduction and the inability of the droplet particle sizes to influence respiratory dust [35,36]. To solve the above problems and to propose reasonable measures, including conduction simulation and calculation of the dust transport law of the return tunnel [37], the use of supersonic spray dust reduction technology is proposed in order to meet the requirements of tunnel resistance and to influence of the tunnel wind flow, efficient capture and dust reduction of small particle size dust [38].

Based on the supersonic siphon pneumatic atomization principle, this paper proposes mobile vehicle-mounted pneumatic spraying combined with full-section fog curtain dust control technology, which is applied on site in the return air tunnel of the 00 comprehensive mining working face at the Min Dong Yi Mine. The application of this technology can effectively solve the problem associated with the deformation of the roadway fog curtain device, which is difficult to fix, through a reasonable dust control technology to solve the problems associated with wind resistance to spray efficiency loss. It provides the relevant theoretical support and means of treatment for the management of dust pollution in the return airway of the underground coal mine.

## 2. Mathematical model

The return air tunnel is a finite space, and when the air velocity at the entrance of the return air tunnel is stable, the dust is diffused and transported in a steady state wind flow. Based on the principles of computational fluid dynamics CFD and discrete element DEM, the method of steady state calculation of wind flow and particle tracking is applied, and the turbulence and particle motion module is used in COMSOL software for modelling and simulation [39,40].

Based on the gas–solid two-phase flow theory [41,42], a systematic admissibility analysis of dust was developed according to Newton’s second law to establish the dust particle dynamics motion in the return air tunnel:

$$m_{p}\frac{du_{p}}{dt}=F_{g}+F_{f}+F_{d}+F_{x}$$ (1)

where *m*_*p*_ is the mass of dust, mg;

***u***_***p***_ for dust velocity, m/s;

***F***_***d***_ is the traction force on the dust, N;

***F***_***g***_ is the gravity of the dust, N;

***F***_***f***_ is the buoyancy of the dust in the air, N;

***F***_***x***_ for other forces, N.

1. Gravity
  The gravity of the dust can be determined by the following equation:
$$F_{g}=m_{p}g=\frac{\pi }{6}d_{p}{}^{3}\rho_{p}g$$ (2)
  where *d*_p_ is the aerodynamic diameter of the dust, m;
  *ρ*_p_ is the density of the dust, kg/m^3^. The proposed dust is coal dust with a certain humidity, and the particle density is set at 1.63 kg/m^3^.
2. Buoyancy
  The dust in the wind stream is subjected to the buoyancy force of the air on it, which is calculated as:
$$F_{f}=m_{g}g=\frac{\pi }{6}d_{p}{}^{3}\rho_{g}g$$ (3)
  where ρg is the density of air, kg/m^3^.
3. Stoke traction
  For dust movement in the airflow by the airflow characteristics of the force on the dust, the trailing force ***F***_***d***_ is:
$$F_{d}=\frac{1}{8}\pi C_{D}\rho_{p}d_{p}{}^{2}|U_{g}‐U_{p}|(U_{g}‐U_{p})$$ (4)
$$Re=\frac{\rho d_{p}|U_{g}‐U_{p}|}{\mu }$$ (5)
$$C_{D}=\frac{24}{Re(Re)}$$ (6)
  where *U*_g_ is the gas velocity, m/s;
  *U*_p_ is the particle velocity, m/s;
  *μ* is the fluid dynamic viscosity, Pa·s;
  *t* is the time, s;
  *R*e is the Reynolds number.
4. Pressure gradient forces
  The dust force due to the pressure gradient in the flow field is calculated as:
$$F_{p}=‐\frac{1}{6}\pi d_{p}{}^{3}\frac{dp}{dx}$$ (7)
  where *p* represents the pressure distribution on the particle surface due to the pressure gradient.

## 3. Physical modelling and meshing

### 3.1 Physical model

Considering that most of the time the workers spend in the mine working is on the pavement in the middle of the stand; thus, the coal dust concentration at the height of the breathing zone of the pavement was chosen as the test site. Through on-site measurement and collection of coal dust mass concentration, dispersion and particle size distribution in the working condition of the comprehensive mining working face and analysis and collation of the actual measurement data, the preliminary understanding of coal dust concentration distribution in the return airway of the comprehensive mining working face is shown in Tables 1 and 2.

**Table 1:** Measured dust concentration in the return airway of the 00 working face.

Sequence	Measuring point	Dust concentration	Dust category
1	Height of the breathing zone in the air return lane at working face 00	87.83 mg/m^3^	Total dust
2	Height of the breathing zone in the air return lane at working face 00	26.60 mg/m^3^	Respiratory dust
3	Height of breathing zone in haulage lane at working face 00	60.33 mg/m^3^	Total dust
4	Height of breathing zone in haulage lane at working face 00	20.89 mg/m^3^	Respiratory dust

**Table 2:** Measured coal dust dispersion data (%) at the East Wing 00 working face of Mindong I Mine.

Grain size (μm)	≤1	≤2.5	≤5	≤10	≤20	≤50	≤100
Return Air Lane Measurement Points	12.03	28.60	52.05	80.75	98.66	100	100

**Fig 2:**
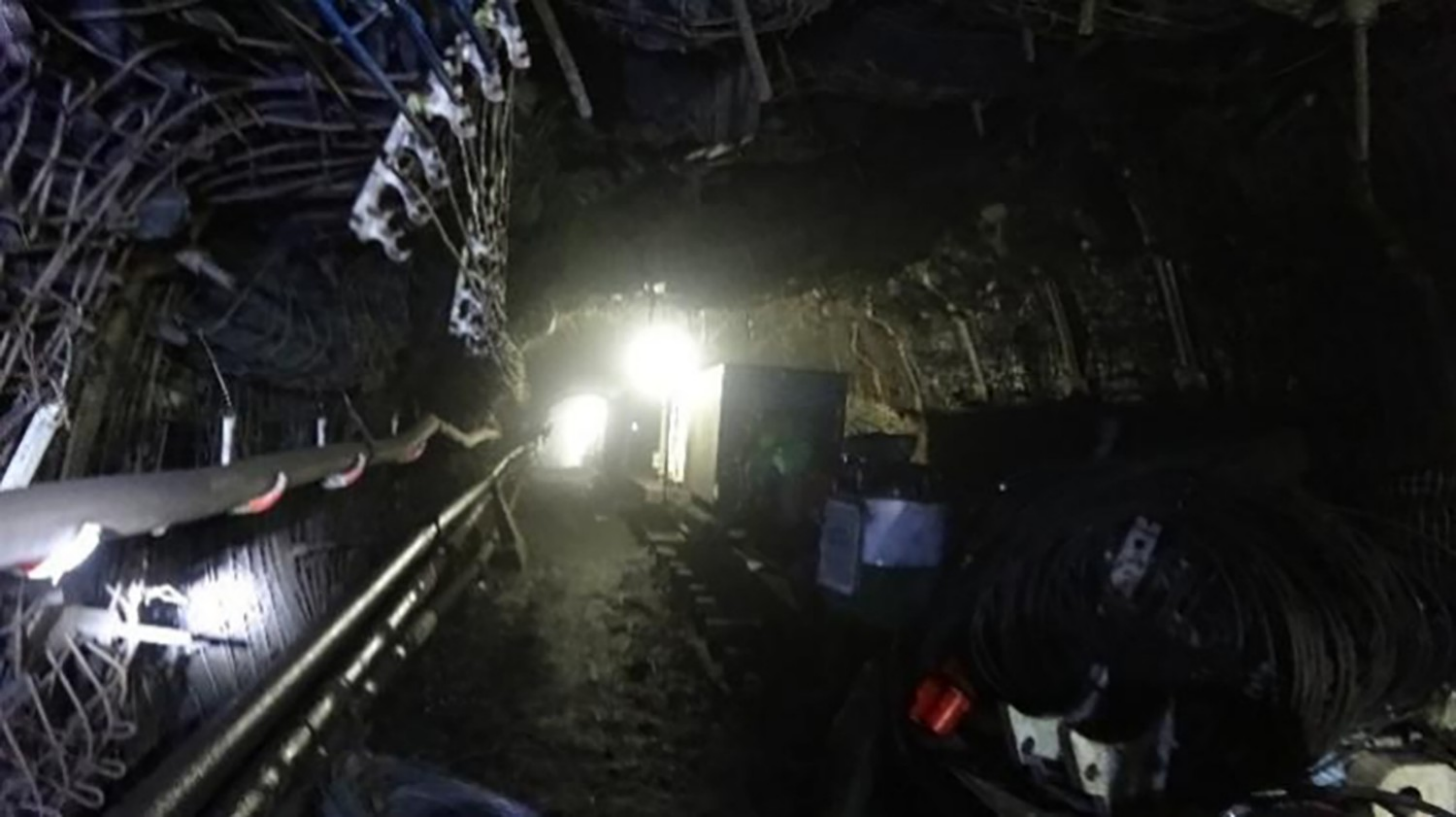
Roadway environment at the return channel.

Fig 2 shows the site environment in the return wind chute, which has a large water content in the coal seam, soft coal rock structure, serious deformation of the roadway roof, floor and sidewalls, narrow roadway space, large changes in the roadway cross-section, large space in the roadway cross-section occupied by mechanical and electrical equipment on trains, and a complex wind flow transport environment, such as empty and full trains, with wind flow velocity in the range of 0.5~1 m/s.

**Fig 3:**
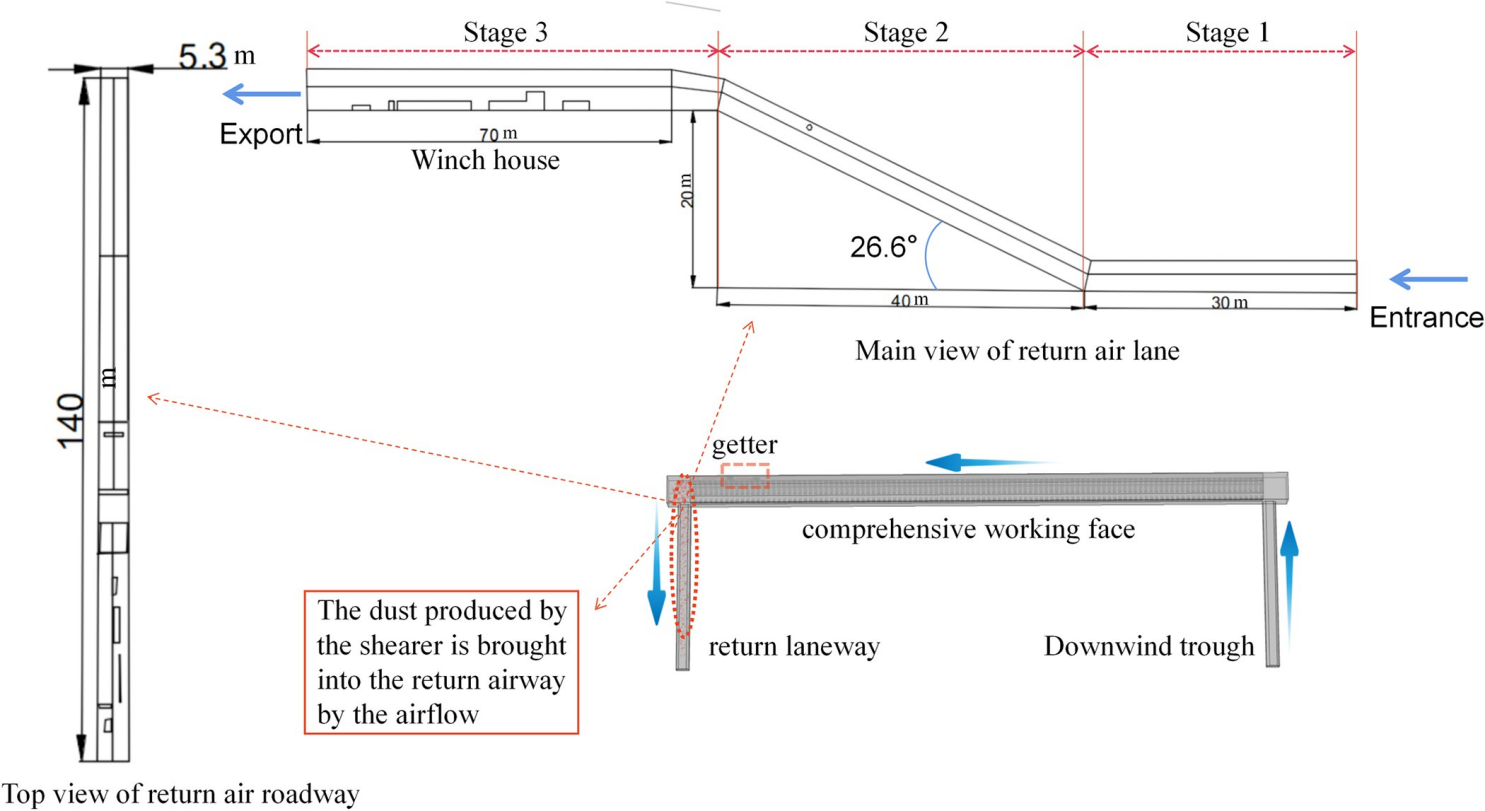
Model of the return air tunnel.

As shown in Fig 3, according to the site situation of Mindong No.1 mine, the geometric model of the return air tunnel of 00 comprehensive mining face was established using COMSOL built-in geometric modelling program; the total length of the tunnel is 150 m, which consists of three stages; the distance from the entrance to the slope of 30 m is the first stage, the slope with an inclination angle of 26.6° and a horizontal length of 40 m is the second stage, and the distance from the exit to the end of the slope of 80 m is the third stage. The third stage is the winch house within 70 m, the winch house section size is 5.3 m x 4 m, the chute section size is 4.8 m x 3.2 m, the lengths are 70 m and 80 m, respectively, the height difference is 20 m, a number of structures are set in the winch house, and the transition position of the tunnel section is smoothly handled by means of a release method.

**Fig 4:**
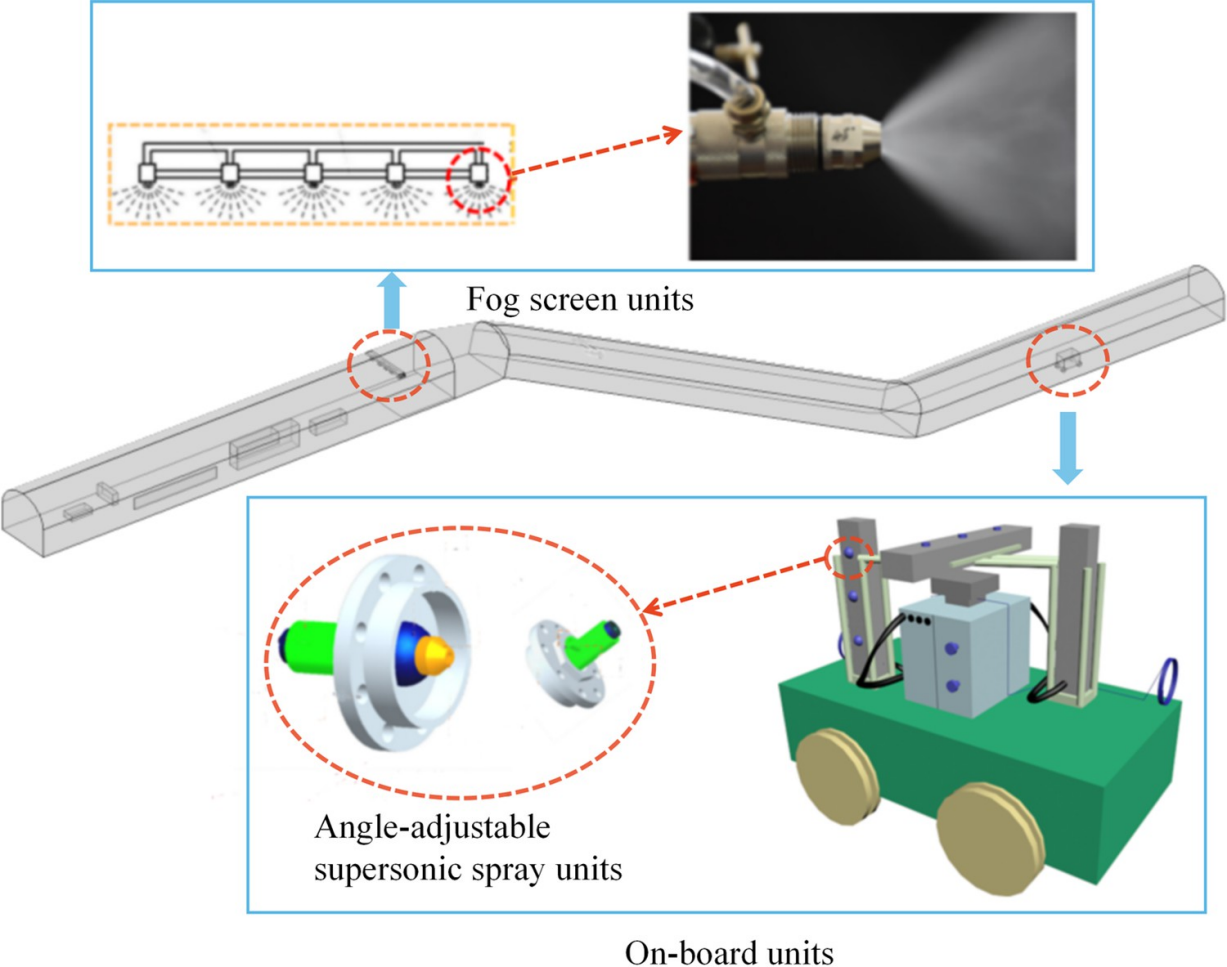
Dust control technology device.

As shown in Fig 4, the dust control technology consists of a mobile vehicle-mounted unit and a fog screen unit, where the full-section supersonic pneumatic fog screen consists of five side-by-side supersonic pneumatic spraying devices that move downwards vertically, and the mobile vehicle-mounted unit consists of nine multiangle supersonic pneumatic spraying devices and the vehicle body, where the nine spraying devices are divided into three groups facing the top and sides of the return tunnel, with three spraying devices in each group at angles of 45°, 0° and -45°.

The device is mainly composed of staggered forms of fixed brackets, supersonic siphon pneumatic nozzles, controllers, multifunctional control boxes for air and water distribution, light control sensors, stainless steel split boxes and other parts. As shown in Table 3, the function of each part is:

**Table 3:** Functions of dust control technical components.

Programme components	Size (mm)	Model	Function
Fixed bracket in a staggered form	4000*600*400		The other components are fixed and supported on the transformer train flatcar and is moved as the vehicle is withdrawn.
Supersonic siphon pneumatic atomization nozzle	Length 80mm, top radius 15mm, bottom radius 22mm	FMMHE-3	The fog screen spraying device in the programme is able to draw clear water from the water tank in the multifunctional control box for air-water distribution by means of self-priming pneumatic processes and is effective in capturing fine dust.
Controller	600*550*400	1747-L542	Connected to 127 V power supply, with functions such as light-controlled spraying and timed spraying, mainly through sensor signals to control the explosion-proof electronically controlled ball valve in the multifunctional control box for air and water distribution to control the work of the nozzle.
Multifunction control box for gas and water distribution	540*500*450	GX-N30Ma	With multiple nozzle air volume distribution control and adjustment functions, the electric ball valve can be short-circuited on the system when the electric ball valve fails, allowing for emergency manual control.
Infrared sensor	Length 65mm, radius 15mm	GX-N30ML	Detection of personnel signals; when the staff passing through the control box can be transmitted to the signal control, the electric control ball valve opens and closes to achieve spray automatic control purposes; the use of a stainless steel split box is mainly to protect the gas and water connection pipeline, for the nozzle to be fixed and support other parts of the composition.
Stainless steel split box	1100*800*750		It mainly protects the air and water connection lines and provides fixed support for the nozzle.

The particle size distribution was tested by the spray laser particle size meter Winner31 at 0.4MPa, 0.6MPa and 0.8MPa, as shown in Table 4.

**Table 4:** Nozzle atomization characteristics.

pressure /MPa	Gas flow rate (m^3^/h)	Water flow rate (L/h)	D_10_	D_50_	D_90_
0.4	3.5	6.1	10.985	30.394	48.306
0.6	4.2	7	9.198	11.431	15.276
0.8	4.4	8.5	8.817	10.752	15.616

The box mainly includes explosion-proof electronically controlled ball valves, pressure reducing valves, manual valves, float control valves, sedimentation tanks, and air and water flow distribution areas and can be equipped with display instruments.

### 3.2 Grid division

**Fig 5:**
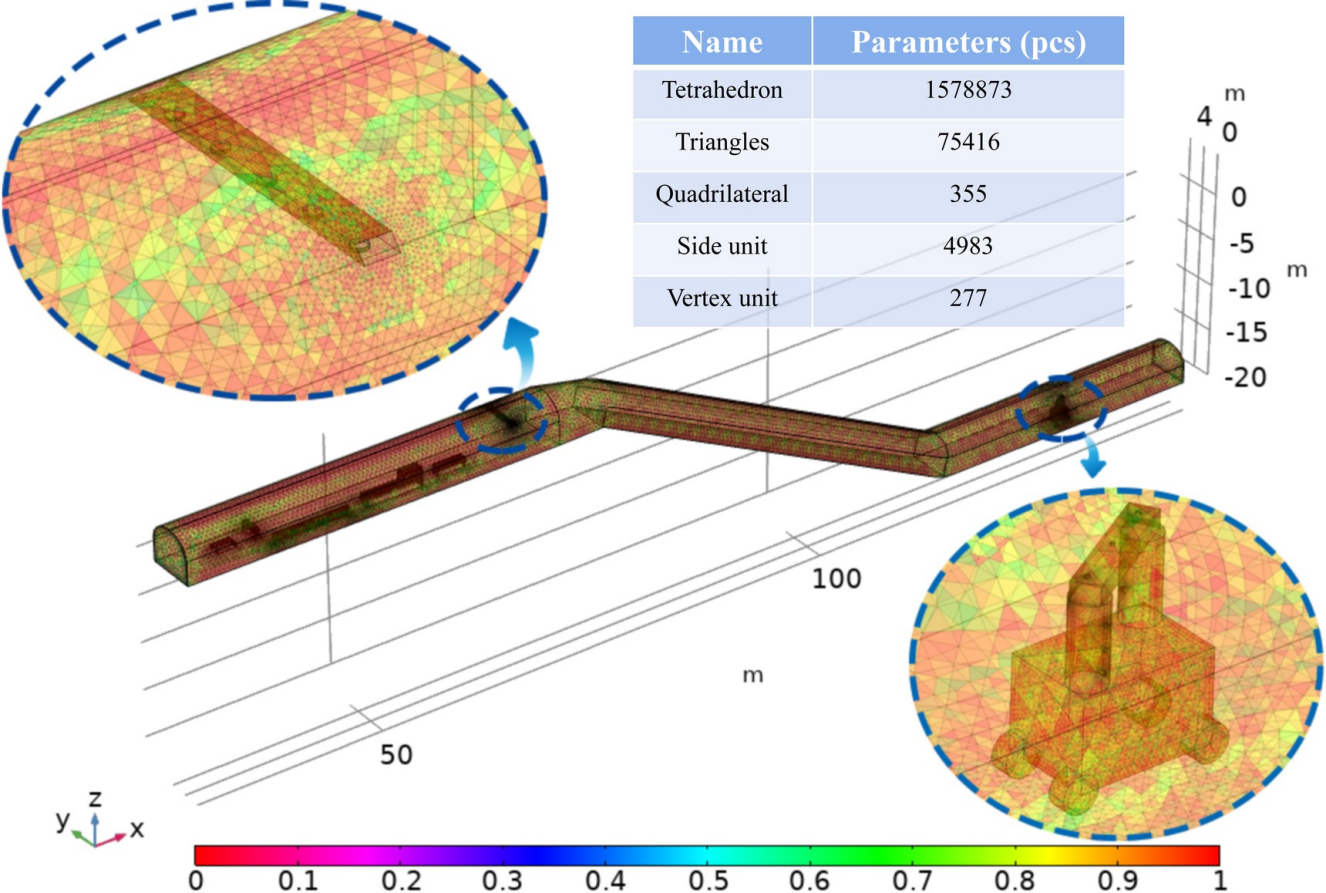
Grid division diagram.

The geometric model was meshed through the grid, and the overall quality of the grid was improved by modifying the size, shape and density of the low-quality grid. The accuracy of the numerical simulation results is closely related to the quality of the grid. Refinement is used at the transition of the section and is also used at the change in height difference angle to establish a total of 19,01345 return air tunnel grids with an average grid mass of 0.21. A dust control technology device grid is established, and the overall grid division is conducted to obtain a total of 2387349 grid cells, of which the minimum cell mass is 0.005064, the average cell mass is 0.6803 and the grid volume is 2025 m^3^, as shown in Fig 5.

### 3.3 Boundary conditions and parameter settings

According to the actual conditions of the return air alley of the 00 header mining face at Mindong I mine, the mesh file was imported into COMSOL, and the boundary conditions and dust source parameters of the geometric model are shown in Table 5.

**Table 5:** Boundary conditions and parameter values.

Project	Name	Parameter setting
Boundary condition	Entrance wind speed (m/s)	0.6
	Outlet pressure (pa)	0
	Air density(kg/m^3^)	1.25
Dust source parameters	Continuous-phase dynamic viscosity (Pa·s)	1.8×10^−5^
	Molecular diffusion coefficient of gas (m^2^/s^-1^)	2×10^−5^
	Density of particles themselves (g/cm^3^)	1.33
	Number of entry particles	1000
	Max particle size of dust (m)	3.16e-6
	Min particle size of dust (m)	1.43e-5
Droplet source parameters	Nozzle flow rate (L/min)	11.5
	Initial velocity (m/s)	10
	Half angle of atomization (°)	40

### 3.4 Reliability verification and analysis

**Fig 6:**
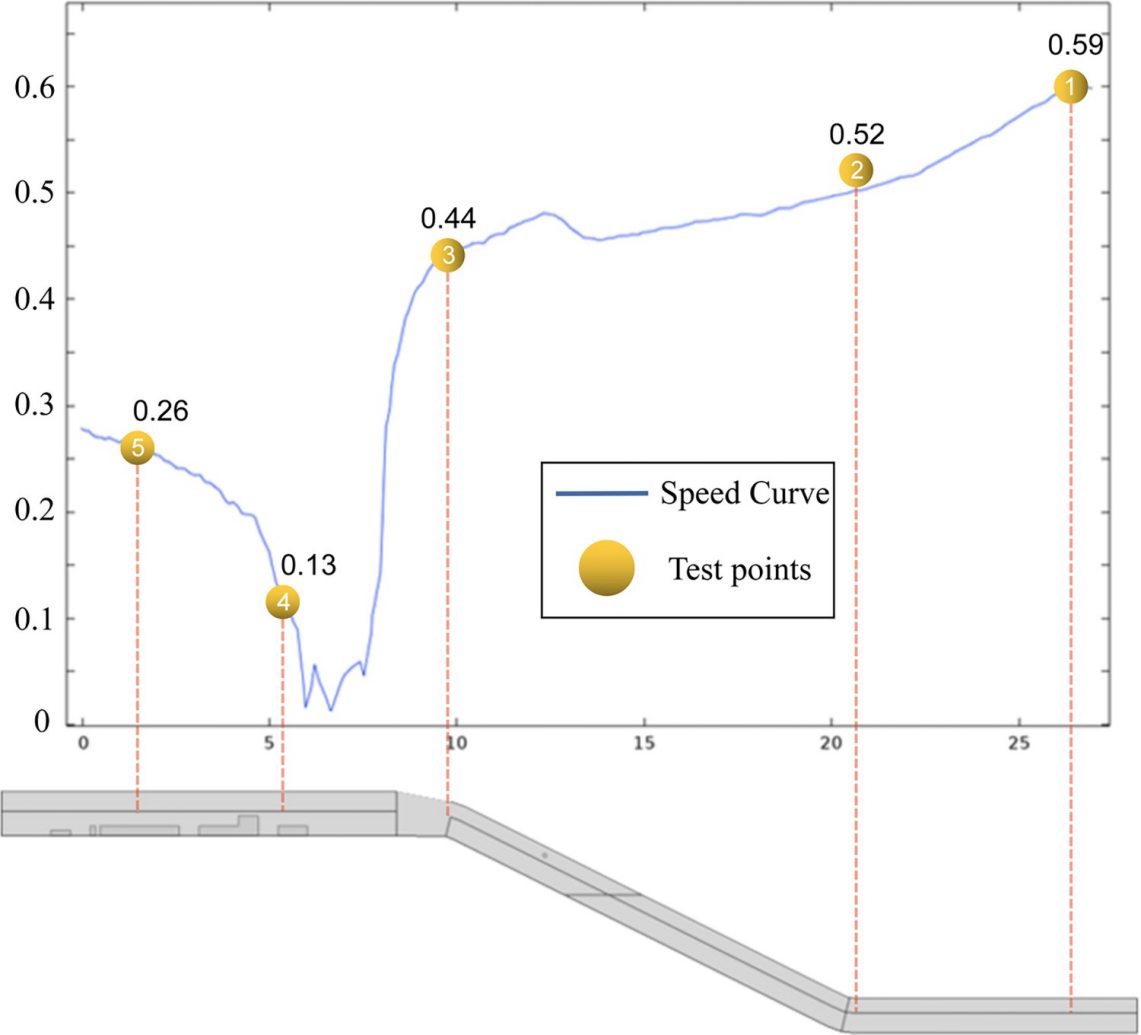
Data comparison diagram.

After the anemometer field wind measurement, the wind speed simulation data were intercepted, as shown in Fig 6. The speed curve and the field test data of the yellow measurement point graph were used for comparison. To verify the correctness of the simulation, the graph of each measurement point wind flow velocity size comparison matched well enough to prove the correctness of the simulation.

## 4. Results and discussion

After setting the boundary conditions and dust source parameters, the algorithm is applied to calculate the steady state and transients, setting the number of iterations to 2000. The data are obtained after convergence. Postprocessing of the simulation results. To clearly analyse the wind flow and dust dispersion pattern in the return air tunnel and the droplet distribution pattern of the dust control technology device, the simulation results are as follows.

### 4.1 Wind flow distribution analysis

**Fig 7:**
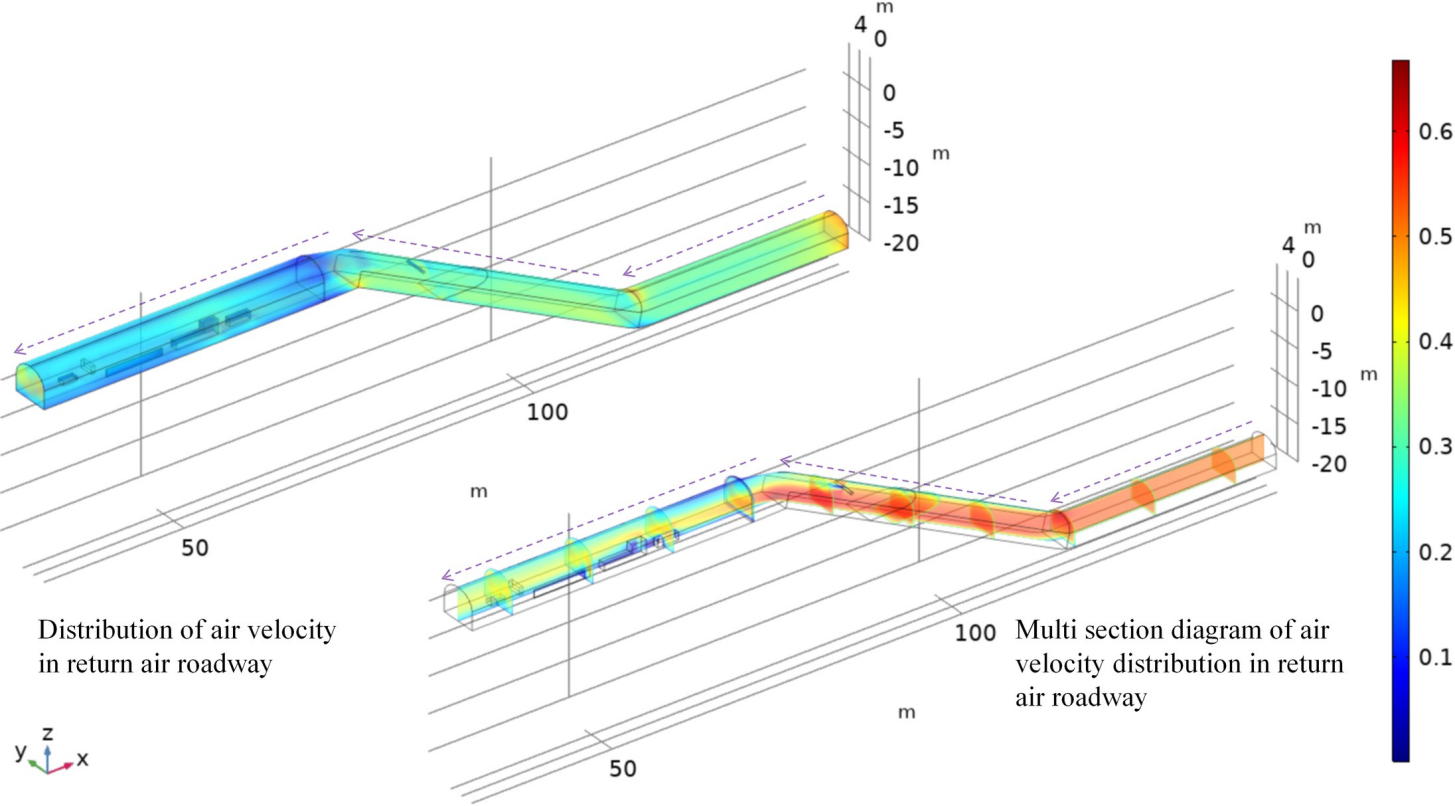
Wind flow velocity distribution in the return lane.

As shown in Fig 7, in the first stage, the wind flow reaches the slope from the entrance of the return air tunnel, forming a wind flow field with a velocity of approximately 0.5 m/s. At the intersection of the first and second stage, the wind flow is influenced by the angle of the slope, forming a small compressed wind flow above the intersection, and the velocity increases to 0.55~0.6 m/s, which is the maximum value in the tunnel. In the second stage, the compressed wind flow at the intersection hits the slope floor, and the overall wind flow momentum continues to drop, reaching the exchange between the second and third stage. The wind flow below the intersection is greater than the wind flow above, and the velocity drops to 0.35~0.45 m/s. In the third stage, the wind flow transitions from a small section to a large section, causing the velocity to drop to 0.25~0.4 m/s, and the velocity of the wind flow on the near wall side is approximately 0.2 m/s. By the winch room and many other structures, compressed wind flow due to local acceleration movement, and structures under the wind side speed are extremely low due to the near wall side resistance, and winch room central wind flow speed of approximately 0.38 m/s, which is a slower around speed. Analysis of the overall transport changes the wind flow rate in the return lane, resulting in increased wind flow in the lane, resulting in the local wind flow above the junction to become compressed and the speed increase, causing the slope to resume horizontal movement, and the local wind flow below the junction by the second compression and to increase speed. The average velocity of the incompressible airflow decreases when the wind flow transitions from a small section to a large section but is influenced by the structure, generating compressed airflow with high velocity in the middle of the tunnel.

### 4.2 Analysis of dust dispersion patterns

**Fig 8:**
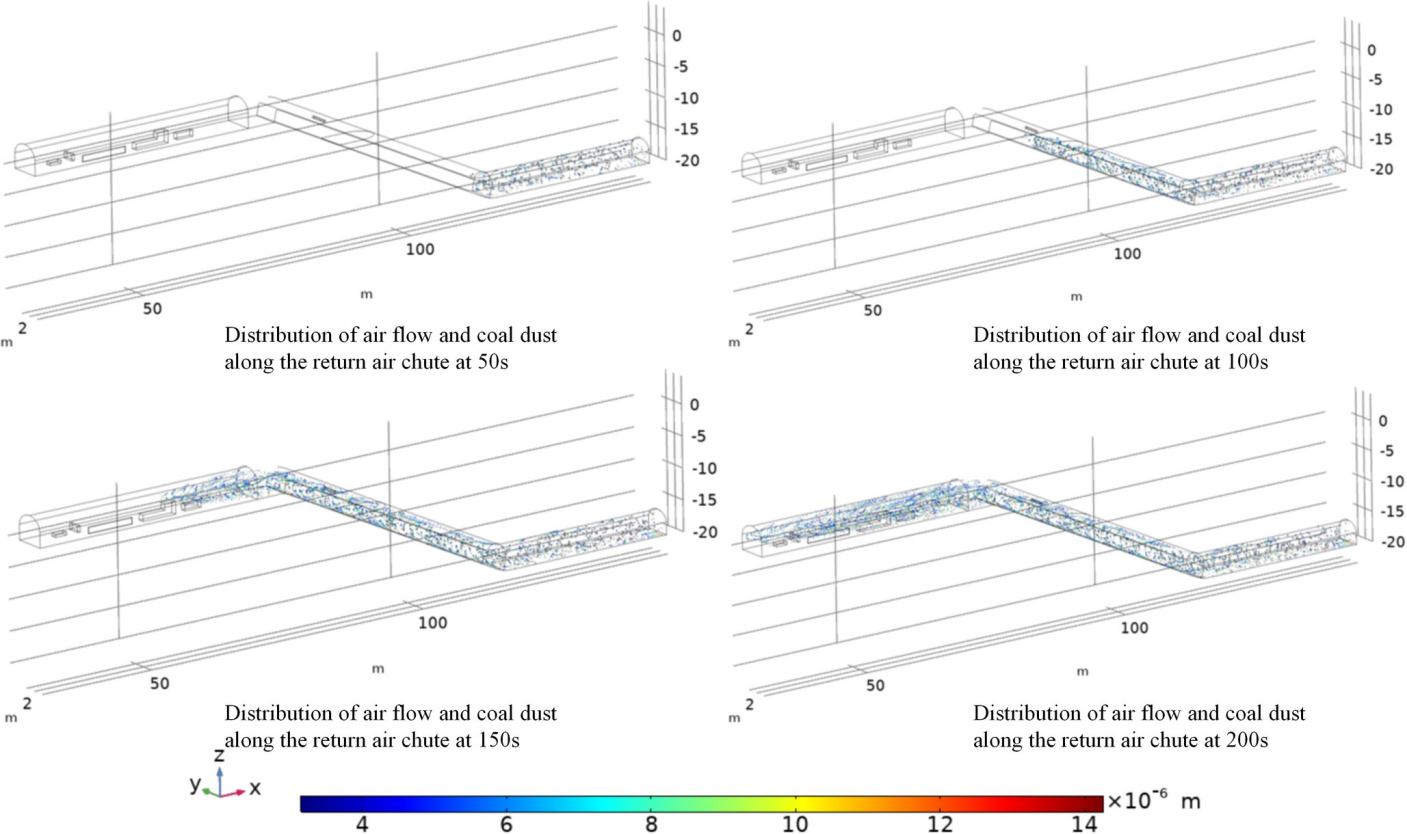
Wind flow in the return wind chute—coal dust distribution pattern.

As shown in Fig 8, at 50 s, the dust spreads out at 30 m, with each particle size suspended in the tunnel and distributed along the flow line. At 100 s, the dust spreads out at 70 m, with each particle size suspended in the lane and distributed along the flow line, adhering to the slope and coalescing at the bottom of the slope. The large dust particles have a high inertia and are prone to freezing on the slope due to inertia forces when the wind flow changes. At a time of 150 s, the dust spreads out at 80 m, and each particle size is suspended in the tunnel and distributed along the wind flow line, adhering to the structure and coalescing at the base of the structure. The large particle size dust has a high inertia and is easily separated from the wind flow line by inertia forces when the wind flow line changes and freezes on the structure. At low velocities, the dust is continuously retained on the downwind side of each structure. When the time is 200 s, the dust spreads and transports to 150 m. Each particle size dust distribution is suspended in the tunnel and distributed with the wind flow extending the flow line, constantly adhering to the structure, coalescing at the bottom of the structure, and transporting in both sides of the structure and in the high wind speed of the crevice. The large particle size dust have a high inertia, allowing for easy movement when the wind flow line change; this is because the inertia force action out of the wind flow line and in the structure indicates freezing, and in each structure the dust will be constantly stagnant and rotate in the low velocity area of the wind flow on the downwind side of each structure.

**Fig 9:**
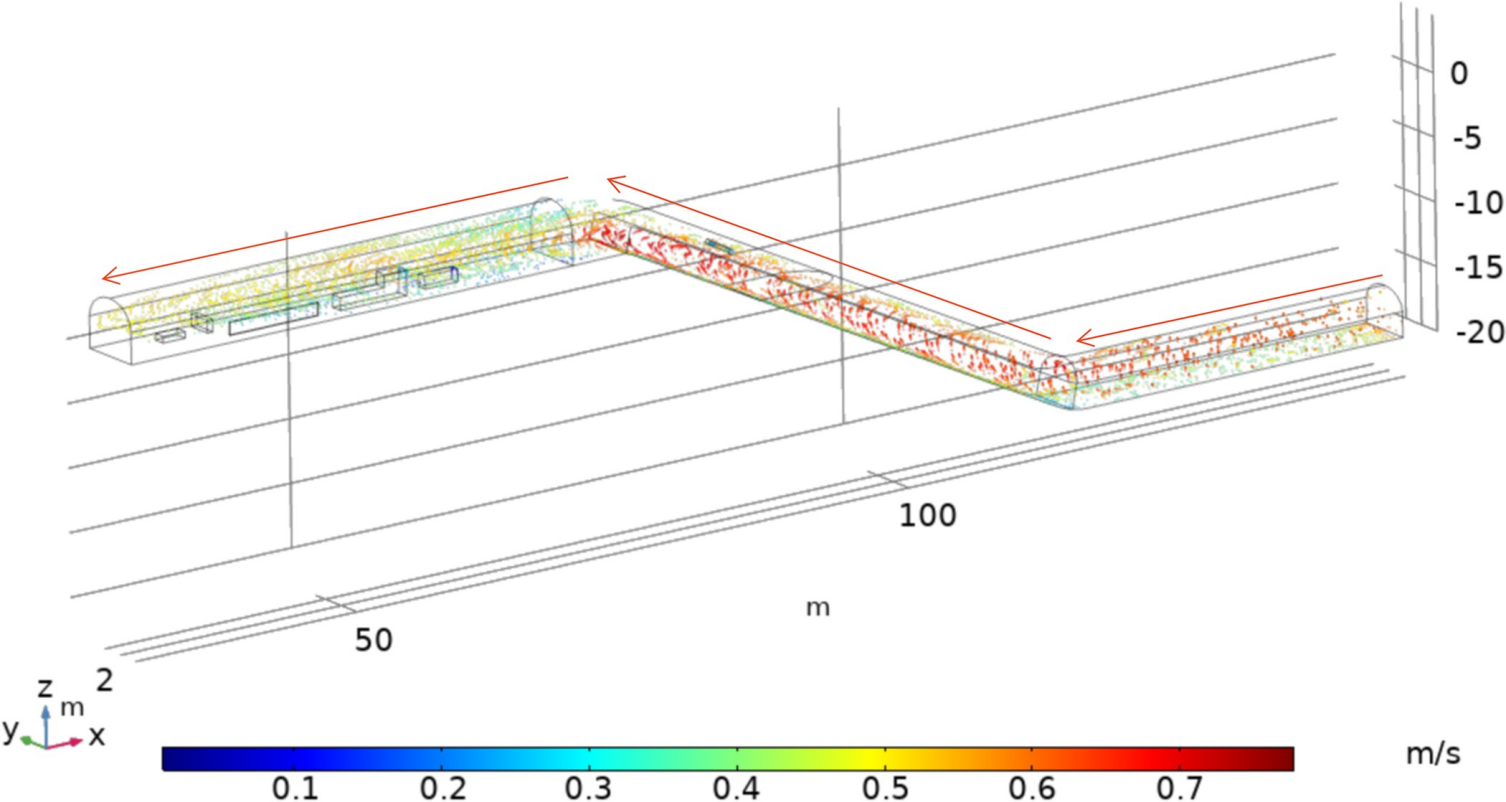
Return wind flow—coal dust velocity distribution.

As shown in Fig 9, the dust velocity and the wind flow velocity remained basically the same at 200 s. The dust velocity in the winch room is between 0.3~0.6 m/s, and the dust velocity in the low elevation tunnel is between 0.5~0.8 m/s. The velocity changes at the structure crevice and section change and the velocity increases at the crevice; this cause the section to expands; because the dust velocity is subject to inertia at first and remains unchanged; due to the sudden drop of the surrounding air velocity, these dusts are affected by the air friction as well as the relative velocity increases the resistance, which gradually reducing the speed and allows for dust accumulation.

According to the analysis of the simulation results, it is found that with the increase in dust transport time, each particle size dust is suspended in the tunnel. After entering the winch room, part of the dust coalesces in the bottom plate of the structure, affected by the compressed wind flow. Dusts are then transported at high wind speeds on both sides of the structure and in the crevice, and large particle size dust inertia is larger. When the wind flow line changes, different particle size dusts affected by the inertia force distribution are distinct. Particle sizes greater than 6.5 μm dusts with particle sizes greater than 6.5 μm accumulate below the structure with velocities in the range of 0.25~0.4 m/s, while dusts with particle sizes less than 4.5 μm are mostly suspended above the structure with velocities in the range of 0.4~0.55 m/s.

### 4.3 Analysis of air-droplet diffusion patterns

#### 4.3.1 Analysis of the airflow distribution pattern of the device

**Fig 10:**
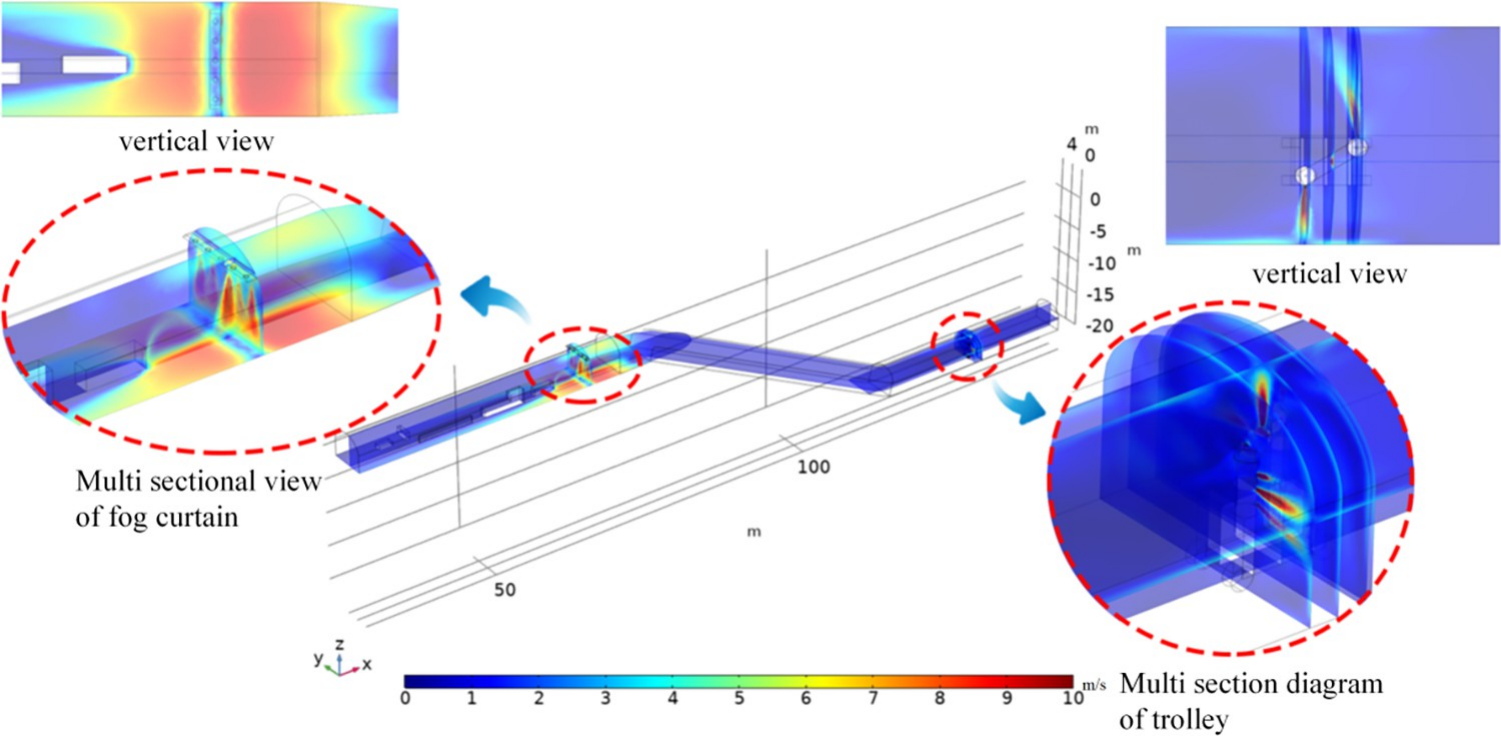
Installation airflow velocity distribution diagram.

As shown in Fig 10, when the dust control technology device is turned on, the maximum velocity of the nozzle air jet can reach 9.8 m/s. The average velocity of the dust control technology device airflow reaching the alleyway wall is approximately 2.1 m/s, indicating that the device’s pneumatic fog screen drives the surrounding airflow and significantly increases the airflow velocity in the return airway. The airflow from the mobile vehicle-mounted fog curtain device is deflected by the influence of the airflow in the lane in nine different directions. The airflow towards the exit of the return lane is blocked by the structure, slowing down and dispersing to the sides of the structure. The airflow towards the slope is influenced by the airflow of the tunnel and creates an interaction force, forming a return flow towards the top of the tunnel with a speed of approximately 4.3 m/s.

#### 4.3.2 Analysis of droplet diffusion velocity patterns

**Fig 11:**
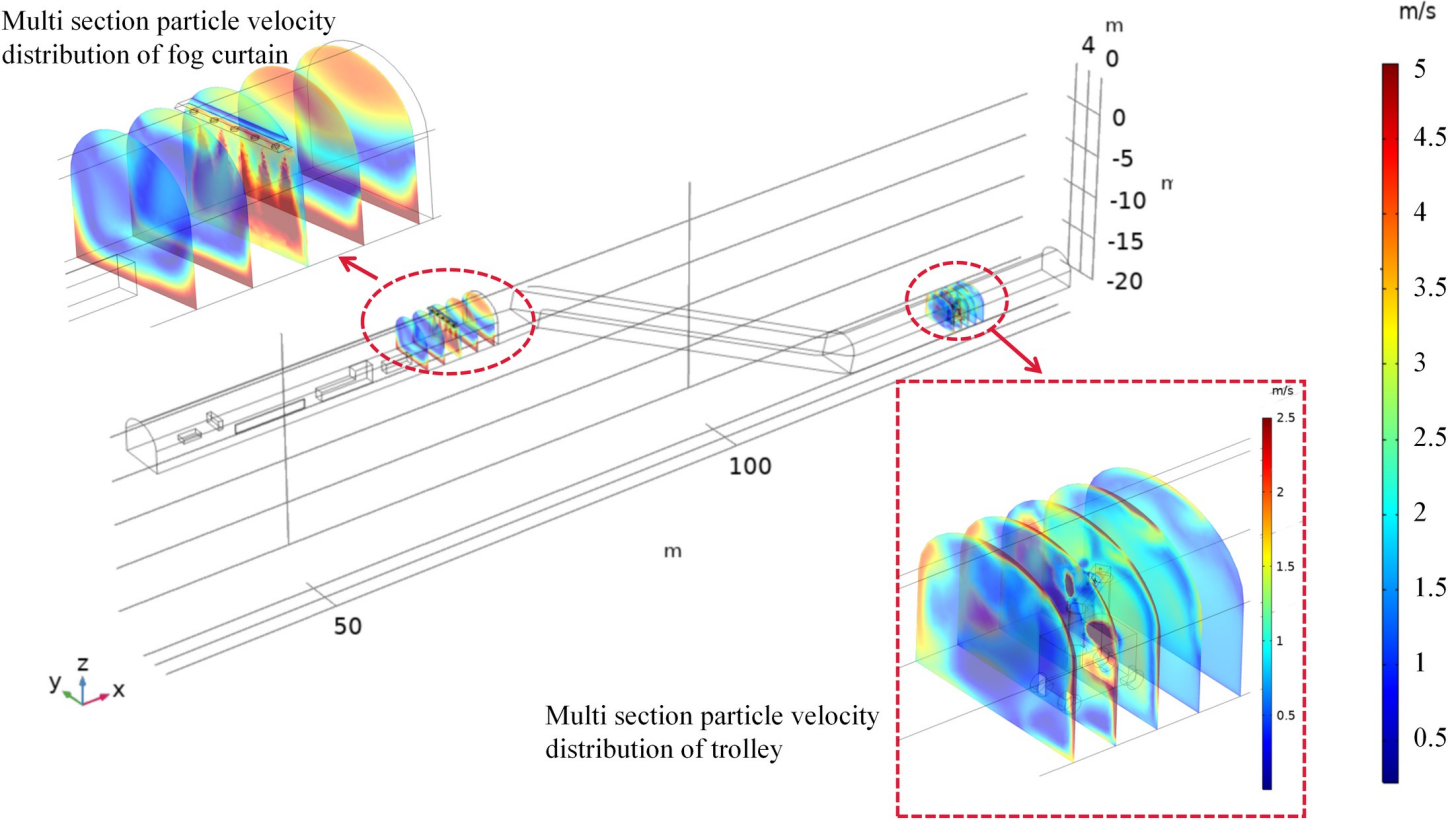
Droplet velocity distribution diagram.

As shown in Fig 11, the droplet movement velocity of the cross section (x = 65.5 m) is ejected from the fog screen device and gradually increases on both sides. Under the influence of supersonic airflow, the droplet gradually spreads to both sides of the fog screen device after impacting the bottom plate of the alleyway. The maximum droplet velocity is 4.8 m/s, the high-speed reflux droplet is mainly concentrated in the range of x = 65.5–68 m, and the average velocity can reach 2.5 m/s. The droplets are influenced by the airflow of the tunnel, moving along the direction of the tunnel airflow diagonally upwardss to the top of the tunnel and gradually spreading to the whole tunnel. Under the combined effect of the airflow and gravity fields, the dust is captured by the high-speed return droplets, and the dust concentration gradually decreases. The droplets within the cross section (x = 129–133 m) are ejected from the mobile vehicle mounted fog screen device, and the movement speed is distributed at different angles, forming a range of coverage, disturbed by the alleyway wind flow. The average speed can reach 1.3 m/s. Due to the influence of the alleyway wind flow, some of the droplets continue to move towards the back of the alleyway with the wind flow, which can effectively suppress the large amount of dust entering the return alleyway, forming a preliminary filter deposition. The remaining dust that is difficult to handle reaches the fog screen and is in the return range for controlled dust reduction.

#### 4.3.3 Analysis of the particle size distribution

**Fig 12:**
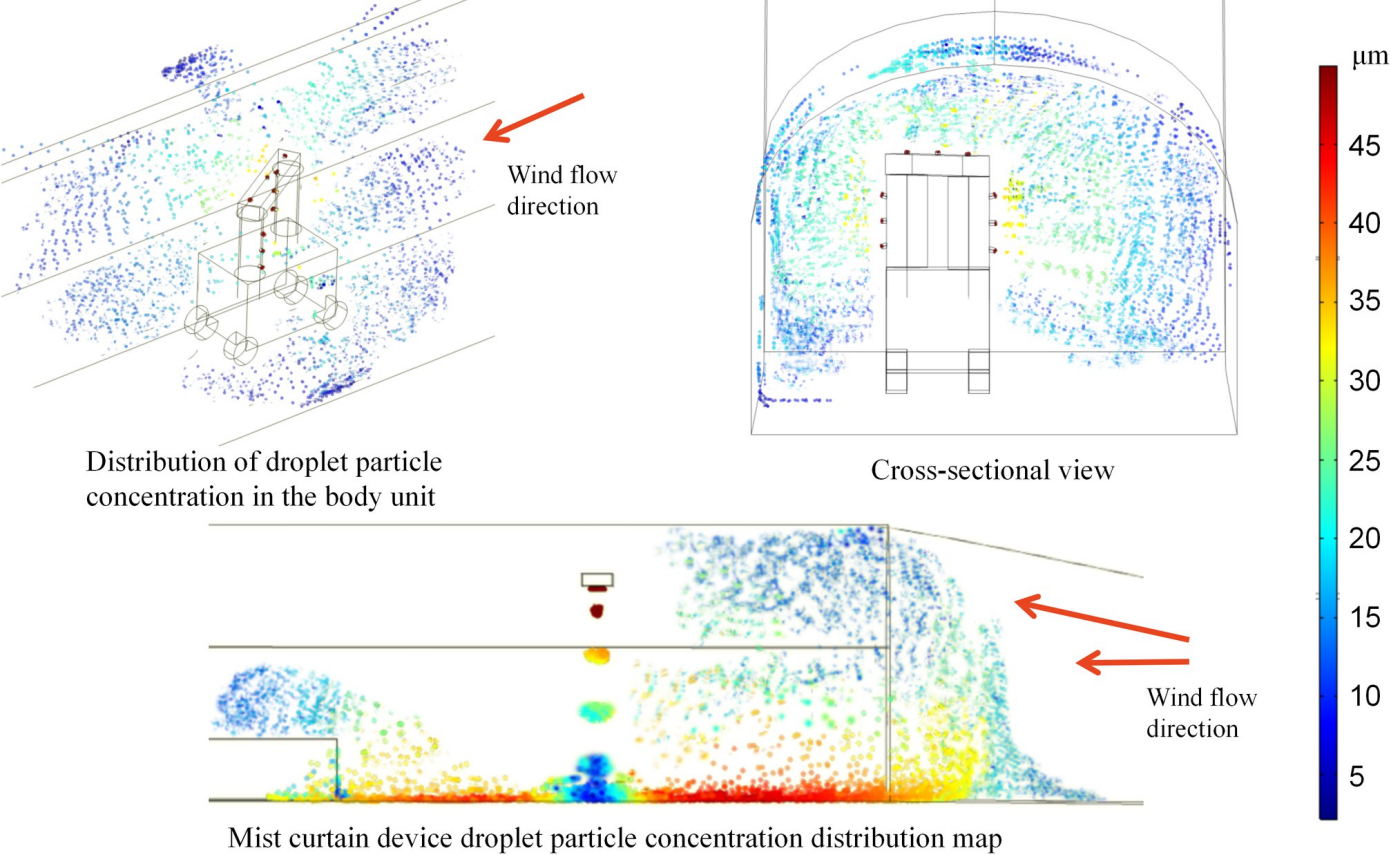
Droplet size distribution map.

As shown in Fig 12, the average droplet size in the return airway is approximately 24 μm when all devices of the dust control technology are switched on simultaneously. At the position of the mobile vehicle-mounted fog screen device, the droplet concentration is high due to the simultaneous action of the nine spray devices, enabling effective dust control at the full cross-section. The droplets are influenced by the tunnel wind flow and collect on the downwind side of the vehicle-mounted unit, forming an area of high concentration. The average droplet size in the range x = 127–130 m is lower than the average droplet size in the range x = 131–133 m. As the distance from the vehicle mounted device increases, the large droplet size gradually settles to the bottom due to gravity, and the droplet concentration gradually decreases. At the position of the fog screen device, the droplet concentration continues to rise due to the simultaneous action of the five spray devices. The droplets are influenced by the airflow of the tunnel and the device and gather on the upwind side of the fog screen device, forming a high concentration reflow area. The return flow position allows for effective control of dust across the entire section, with droplets larger than 25 μm suspended and deposited below the lane and droplets smaller than 25 μm suspended above the lane. The average droplet size in the range x = 62 to 65.5 m is lower than the average droplet size in the range x = 65.5 to 68 m. The droplets on the downwind side of the mist screen device are influenced by the structure, with the larger droplets deposited at the bottom of the structure and in the gaps and the smaller droplets suspended above the structure and moving towards the exit of the return lane under the influence of the lane wind flow.

## 5. Engineering application

**Fig 13:**
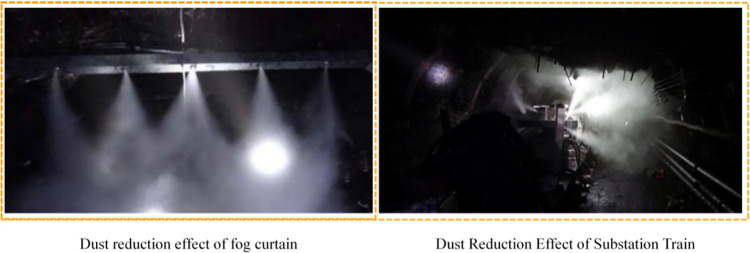
Site treatment results.

The dust control technology forms the spraying effect, as shown in Fig 13 below. Considering the influence of uncertainty factors, the dust concentration measurement was started when the site dust concentration reached the maximum at 20 minutes of production at the site working face, and the site tunnel wind flow velocity was between 0.3m/s~0.55m/s. The measured wind speed of this technology section is in the range of 1.5~2.2m/s, realizing full section spraying, and the fog screen can penetrate the wind resistance to reach the wall of the tunnel under 0.6m/s lateral wind flow. The dust control effect is good, and the actual energy consumption of the program is low, with water consumption of 0.3L per minute and air consumption of 480L.

**Fig 14:**
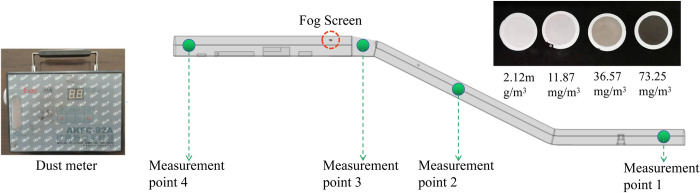
Site plan of measurement points.

The numerical simulation results show that the droplet concentration within the range of the dust control technology device significantly exceeds the dust concentration. To further illustrate whether the corresponding droplet concentration in the space has a reasonable dust raising effect on the dust in the return airway, four measurement points before and after the dust control technology device were selected. As shown in Fig 14, the AKFC-92A mine dust sampler was used for field measurements when the flow field in the working space was relatively stable.

The test results of the actual data on site in the return air lane are shown in Table 6. The concentration of respiratory coal dust pollution at each measurement point in the return air lane of the working face is between 8.19~18.31 mg/m^3^. Through technical treatment to reduce dust, the concentration of respiratory coal dust is 0.93 mg/m^3^, calculated by the formula of efficiency = (original dust concentration—present dust concentration)/original dust concentration, the efficiency of respiratory dust and total dust control reached 97.93% and 96.53%, respectively.

**Table 6:** Comparison of coal dust concentration before and after treatment in the return airway of the 00 working face.

number	Total coal dust concentration/(mg/m^3^)			respirable dust concentration/(mg/m^3^)		
	Total coal dust concentration before treatment	Total coal dust concentration after treatment	Dust efficiency/%	Respiratory dust concentration before treatment	Respiratory dust concentration after treatment	Dust efficiency/%
1	86.82	76.13	12.31	18.31	17.25	5.79
2	71.68	23.93	66.62	14.35	4.84	66.27
3	58.35	20.14	59.49	8.41	2.42	71.22
4	36.41	1.97	94.59	8.19	0.93	88.64
entirety	86.82	1.97	97.93	18.31	0.93	94.92

## 6. Conclusion

In this paper, a dust control technology is proposed for the return air tunnel of a comprehensive mining face. A simulation model is constructed for CFD numerical simulation and combined with practical application in the field, and the following conclusions are obtained.

1. With the increase in wind flow-dust transport time and the influence of the slope, the overall velocity of the tunnel wind flow drops to approximately 0.48 m/s after entering the slope and to approximately 0.39 m/s after leaving the slope. Different particle sizes of dust are distributed differently by the inertial force; dust with particle sizes greater than 6.5 μm accumulates below the structure with velocities in the range of 0.25~0.4 m/s, and dust with particle sizes less than most of the dust with a particle size of less than 4.5 μm is suspended above the structure with velocities in the range of 0.4~0.55 m/s.
2. With the increase in airflow-droplet diffusion time, the average velocity can reach 1.3 m/s. The average droplet particle size in the return lane is approximately 24 μm, droplets with particle sizes larger than 25 μm are suspended and deposited below the lane by the airflow field and gravity field, and droplets with particle sizes smaller than 25 μm are suspended above the lane. The area of high concentration is formed in the range of x = 62~65.5 m and x = 127~130 m, which can achieve effective control of dust in the whole section.
3. After the application of joint dust control technology treatment, the dust concentration in the return air lane of the 00 header mining face of Mindong No. 1 Mine was significantly reduced, with the total dust mass concentration reduced from 86.82 mg/m^3^ to 1.97 mg/m^3^ and the respiratory dust concentration reduced from 18.31 mg/m^3^ to 0.93 mg/m^3^, with dust removal efficiency reaching 97.93% and 94.92%, respectively, and the dust particles were effectively controlled.
